# Supplementary material for: Are carbon emissions trading and green financial instruments synergistic? -Comprehensive quantitative research based on content analysis
Source: PLoS One. 2024 Mar 7;19(3):e0298601. doi: 10.1371/journal.pone.0298601 (PMC10919601; doi:10.1371/journal.pone.0298601)
Supplement: S1 Data — (ZIP) [file pone.0298601.s001.zip › Data/地级市Z_F工作报告.zip!/╜¡╦╒/╒≥╜¡╩╨/└┤╫╘╣1⁄2╓┌║┼í╛┬φ┐╦╩2╛▌═°í┐_2011.docx]

各位代表：

　　现在，我代表镇江市人民政府向大会作工作报告，请予审议，并请市政协委员和其他列席人员提出意见。

　　一、2010年和“十一五”回顾

　　过去的一年，在省委、省政府和市委的正确领导下，全市上下深入贯彻科学发展观，按照“跨越发展、创新驱动、绿色增长、和谐共享”的总体要求，创新克难，锐意进取，全面完成了市六届人大三次会议确定的各项目标任务。预计全市实现地区生产总值1957亿元，比上年增长13.8%；地方财政一般预算收入138亿元，增长36%；全社会固定资产投资1327亿元，增长31.3%；社会消费品零售总额559.5亿元，增长18.9%；实际利用外资16.1亿美元，增长12.1%；城市居民人均可支配收入23075元，增长10.1%；农民人均纯收入10800元，增长12%；城镇登记失业率2.3%；单位GDP能耗下降4.3%，化学需氧量削减2.6%，二氧化硫排放量削减4%。全社会固定资产投资、社会消费品零售总额等指标增幅居全省前列。

　　（一）转型升级迈出大步

　　新兴产业增势强劲。出台新兴产业发展规划纲要，全市新兴产业完成投资227.2亿元，增长106.1%；实现销售1100亿元，增长45%。优势产业扩量升级。装备制造等五大主导产业实现销售3350亿元，丹徒重型装备等3个园区成为省特色产业基地，新增3家销售超百亿元企业；完成技改投资650亿元，增长27.9%。科技创新力度加大。高新技术产业产值占比达36.1%，全社会研发投入占比达2%，科技进步贡献率达53%，年专利申请量突破万件，成为国家“知识产权工作示范市”和“知识产权质押融资试点城市”。现代服务业亮点频现。服务业增加值占比达40%。软件产业营业收入103亿元，增长56.5%；服务外包执行额1.6亿美元，增长159.4%；文化产业完成投资36.7亿元，增长102.6%；镇江港建成亿吨大港，惠龙国际钢铁物流交易额突破100亿元；商业城、八佰伴零售额均超10亿元；新增私家车4.6万辆，增长50.2%。高效农业快速发展。新建12个市级现代农业园区，句容现代农业产业园升格为省级园区。“三资”投入农业40亿元。新增高效农业面积30万亩、农村“三大合作”组织412家、入社农户7.8万户。

　　（二）要素集聚成效明显

　　镇江经济技术开发区、大学科技园、京口软件园和润州特种船舶基地晋升为国家级；镇江科技新城建设快速推进；成功引进11家省以上研发机构和检测中心，成立省航空新材料等4个产业技术创新战略联盟，新增5家企业院士工作站，新建孵化器面积超过100万平方米。新引进38个人才（团队），3人入选国家“千人计划”，28人进入省“双创”计划。12个央企项目落户，总投资322亿元；净增私个注册资本超1000亿元，增幅全省第一。发行企业债券76.3亿元，总量全省第一。中信银行、紫金保险分支机构落户，成立江苏银行镇江科技支行，新增2家村镇银行、11家小额贷款公司，金融生态县实现全覆盖。

　　（三）城乡面貌变化显著

　　全市完成城建投资375亿元、拆迁779.4万平方米。新行政中心建成，体育会展中心“两馆”封顶，“三山”改造和金山湖景观工程基本完成，苏宁广场、万达广场等一批城市综合体加速推进，大市口地下人防工程主体完工，完成老小区改造50万平方米。交通基础设施投入90亿元，创历史新高。沪宁高铁镇江段建成通车，综合交通客运枢纽开通运营，京沪高铁、宁杭高铁、泰州大桥及东接线、338省道镇江段建设进展顺利；开通大运量公交3号线，建成丹徒新城等一批公交停车场。11个试点新市镇、50个试点新社区建设进展良好。全社会环保投入73.9亿元，市区通过国家环保模范城市省级复核，丹阳、句容、扬中和丹徒通过国家生态县省级技术评估，33个镇通过国家生态镇省级考核。城市水利建设走在全国前列，基本解决农村饮用水安全问题。建成区绿化覆盖率42.4%，全市森林覆盖率21.5%。

　　（四）社会事业加快发展

　　全市财政用于民生投入66.4亿元、增长33%，高出一般预算支出增幅5.4个百分点。新增城镇就业9.2万人，转移农村劳动力2.7万人。推进社保制度全覆盖，新农保加快制度接轨，征地保障实施即征即保，30多万农民享受医保“同城同待遇”。完成廉租房、公共租赁房、经济适用房、危改房和拆迁安置房等“五房”建设300万平方米。公共文明指数测评列全国第31位。启动学前教育体制改革国家级试点，推进义务教育优质均衡发展。组建市文广集团和报业集团，西津渡文化创意产业园等项目加快推进，文化惠民活动深入开展。在十七届省运会上获得52枚金牌，首次跻身省金牌奖励榜。公立医院改革进展顺利，完成14家社区卫生服务中心标准化建设，率先实现基本药物制度全覆盖。人口出生缺陷发生率降至4.7‰。积极化解社会矛盾，有效解决信访突出问题。平安创建成效显著，安全生产事故起数和死亡人数连续九年“双下降”，食品药品、产品质量安全水平稳步提高。外事、侨务、对台事务、统计、民族宗教、地方志、档案、防震、老龄、妇女儿童、关心下一代、慈善和残疾人等事业得到加强。国防教育、人民武装、双拥、国家安全工作取得新成绩。对口援建四川地震灾区三年任务两年全面完成，援藏援疆各项工作有序推进。

　　（五）自身建设继续加强

　　新一轮市县、乡镇政府机构改革顺利完成，事业单位改革工作稳步推进。城市社区、农村“两委”换届工作全面结束。自觉接受市人大法律监督、工作监督和市政协民主监督，办理人大代表建议301件、政协提案492件。充分听取和吸纳各民主党派、工商联、无党派人士以及社会各界的意见和建议。依法行政和廉政工作扎实推进，“五五”普法目标顺利完成，行政权力网上公开透明运行全面实施。公共财政建设加强，审计监督力度加大，财政资金使用效益进一步提高。市政府“12345”公开电话接听群众来电22万余次，及时解决热点难点问题。“中国镇江”政府门户网站获全国绩效评估第17名。

　　各位代表，过去一年为“十一五”发展划上了圆满句号。五年来，我们勇于开拓创新，奋力攻坚克难，引领全市人民共建美好家园。今日的镇江，山清水秀、生机勃勃，人心思上、和谐稳定！

　　这五年，综合实力显著提升。经济社会发展主要均量指标、科技综合实力居全省前五，跻身福布斯中国大陆最佳商业城市第33位。同“十五”末比，GDP实现翻番、人均超过9000美元，一般预算收入增长1.9倍，全社会固定资产投资增长2.3倍，社会消费品零售总额增长1.3倍。

　　这五年，发展活力不断增强。277户市属企业改革任务基本完成；29个央企项目落户，总投资872.3亿元；实际到位外资60.5亿美元；民营经济增加值占比达57%；新增上市公司10家、创投公司7家。率先试点中小企业融资新模式，成立全省首家农村小额贷款公司，成功引进6家银行分支机构。

　　这五年，城乡面貌发生巨变。累计完成城乡建设投资超过1000亿元，拆迁总面积1950万平方米，其中市区突破1100万平方米。“三铁一桥”建设开启同城时代，“南城北水”建设全面推进，“青山绿水”工程加快实施，建成区面积扩大22平方公里，奠定了山水花园城市的基本形态。各辖市城市化进程加快，中心城区综合承载能力明显增强。新市镇、新社区、新园区建设完成投资超100亿元，建成农民集中居住区612万平方米，农村社区综合服务中心实现全覆盖，“万顷良田”建设全省领先。

　　这五年，人民生活加快改善。城乡居民收入保持两位数增长，人均储蓄增长近1倍，消除了农村人口绝对贫困现象。实现全面小康省定目标值。荣获首批“中国创业之城”称号。累计新增城镇就业23万人，城镇登记失业率控制在3.5%以内。覆盖城乡的社会保障体系和救助体系基本建立。

　　这五年，社会事业全面进步。实施科教兴市、人才强市战略，连续荣获“全国科技进步先进市”称号，率先基本实现教育现代化。全国公立医院改革试点有序推进。“大爱镇江”等成为镇江文化新品牌。第五次蝉联“全国双拥模范城”，获得“全国社会治安综合治理优秀地市”、“科技强警示范城市”、“中国最关爱民生的城市”和“中国十佳和谐可持续发展城市”等荣誉称号。

　　各位代表，过去五年我们所取得的成绩，是省委、省政府和市委坚强领导的结果，是市人大、市政协监督支持的结果，是全市人民团结奋斗的结果，也与历届领导打下的良好基础分不开。在此，我谨代表市人民政府，向全市人民，向各位人大代表、政协委员和离退休老同志，向市各民主党派、工商联、无党派人士、人民团体和社会各界，向驻镇解放军、武警官兵和公安干警，向垂直部门和驻镇单位，向所有关心支持镇江发展的海内外朋友，致以崇高的敬意和衷心的感谢！

　　各位代表，我们也清醒地认识到，镇江的发展还存在一些深层次问题：整体实力需要进一步增强，支撑转型升级的重大项目推进不快；少数城建项目进展较慢，城市的功能和品质还需要进一步提升，统筹城乡发展的任务还很艰巨；地方财政一般预算收入总量偏小，教育、医疗、住房、收入、保障等民生问题需要继续改善。这些问题，我们将在今后的工作中采取有力措施，认真加以解决。

　　二、“十二五”展望

　　根据市委五届十一次全会审议通过的《关于制定镇江市国民经济和社会发展第十二个五年规划的建议》，市政府编制了《镇江市国民经济和社会发展第十二个五年规划纲要（草案）》，提请大会审议。

　　未来五年，虽然我们将面临诸多不确定因素和各种新情况，但总体上仍是我市跨越发展的重要机遇期、转型升级的关键攻坚期、城乡一体化的加速提升期和人心求富思变的安定发展期。我们将坚持“与苏南其他城市同步迈入基本现代化”的目标定位，走有镇江特色的科学发展路径，到2015年，全市GDP在2010年基础上实现翻番，力争达到4000亿元，年均增长12%以上，人均GDP接近2万美元；五年累计全社会固定资产投资超1万亿元，年均增长18%以上；地方财政一般预算收入超过350亿元，年均增长20%以上；社会消费品零售总额年均增长18%以上；城乡居民收入实现翻番；完成省政府下达的节能减排任务；城市化率力争达到70%。

　　“十二五”期间，我们将奋力拼搏，把镇江建设成为：

　　产业发达城市。着力构建现代服务业、先进制造业与现代高效农业为主体，技术领先、资源节约、环境友好的现代产业体系。

　　创业创新城市。全社会形成创业创新的内生动力、保障机制和人才支撑，建成国家级创业型城市和创新型城市。

　　山水花园城市。中心城区形成“一体两翼多中心”的新格局，呈现“山在城中、城在园中、房在绿中、人在景中”的新局面。推进城乡规划、产业布局、基础设施、公共服务和就业社保五个一体化，展示环境优美、交通便捷、欣欣向荣的乡村新貌。

　　幸福和谐城市。进一步增强全市人民的归属感、荣誉感和幸福感，让城乡大地处处充满爱意、彰显文明、涌动幸福。争创全国文明城市、联合国人居环境奖，率先实现教育现代化、社会保障“双覆盖”。城乡居民“劳有所得”、“住有宜居”、“学有优教”、“病有良医”、“老有善养”，生活质量大幅度提高。

　　三、奋力做好2011年工作

　　2011年是“十二五”的开局之年，做好今年的政府工作至关重要。我们将认真贯彻中央、省有关会议和市委五届十一次全会精神，坚持科学发展主题，突出转型升级主线，致力强市富民主要任务，满怀信心、抢抓机遇，应对挑战、团结拼搏，确保开好局、起好步。

　　全市经济社会发展的主要预期目标为：GDP增长12%；地方财政一般预算收入增长20%；全社会固定资产投资增长20%，实际利用外资增长8%，外贸进出口总额增长15%，社会消费品零售总额增长18%；城市居民人均可支配收入增长12%，农民人均纯收入增长12%，居民消费价格总水平控制在4%左右，城镇登记失业率控制在3.5%以内；全社会研发投入占比达2.1%，高新技术产业产值占比达38%，服务业增加值占比达41.5%；单位GDP能耗下降3.5%，主要污染物排放削减率完成省下达任务。

　　工作中，我们将全力抓好六个方面：

　　（一）坚持以人为本理念，满足人民群众的生活保障需求

　　大力扶持创业。加快培育新时期创业文化，增强市民的求富意识和风险精神，建成国家创业型城市。新发展私营企业6000户、个体工商户9000户，新增注册资本500亿元。继续完善四级创业指导服务体系，成功扶持创业4000人、带动就业3.5万人。着力提高居民工资性、经营性及财产性收入，建立健全农民增收长效机制，努力扩大中等收入群体，不断加快富民步伐。

　　广开就业门路。完善人力资源市场配置机制，基本建成“充分就业市”。新增城镇就业4.3万人、转移农村劳动力3万人，下岗失业人员再就业2万人；接收本科毕业生1万名以上，新培养紧缺型高技能人才5000名，本地大中专毕业生就业率90%以上。创造更多的公益性岗位，为大龄和缺少技能人员、“农转居”新市民等提供就业机会。

　　提高保障水平。完善保障体系，将灵活就业人员、被征地农民等纳入城镇社会保障范围。狠抓社保扩面征缴，确保基本养老、医疗、失业保险覆盖率稳定在95%以上。健全城乡低保自然增长机制和困难群体物价补贴响应机制，切实保障困难群体的基本生活。加大住房保障力度，建设拆迁安置房6.4万套、公共租赁住房1万套、经济适用住房3000套、廉租住房400套。推动老龄服务体系建设，城乡居家养老服务站点覆盖率分别达80%和30%。大力发展慈善事业，推进城乡社会救助体系建设。

　　（二）构建现代产业体系，满足人民群众的物质增长需求

　　推进现代服务业快速发展。认真落实现代服务业两年翻番政策，力争营业收入增长50%以上。加速提升现代物流业，建成2个交易额超100亿元的物流基地。完成新兴商贸业发展规划，高标准打造“一主五副”商贸中心，切实推进14个城市综合体和2个电子商务产业（示范）园建设。新增软件类企业100家。推进国家级“智慧旅游”服务中心建设，争创1家5A级旅游景区和1家五星级旅游饭店。引进1-2家股份制银行，组建2家村镇银行、10家农村小额贷款公司，发展5家以上股权投资机构，力争成为省债券融资试点地区。

　　推动新兴产业爆发式增长。五大新兴产业实现销售1600亿元，增长50%以上，占规模以上工业比重超过33%；当年投资力争翻番。以争创国家级创新型试点城市和全国科技进步先进市“八连冠”为抓手，集中60%以上市级科技经费实施新兴产业重大科技专项。新兴产业科技研发投入占比达到3%以上，引进培育研发检测机构10个以上，高标准建设镇江科技新城，新增各类孵化器面积50万平方米以上。坚持人才优先发展战略，实施新一轮创新创业领军人才集聚工程，引进30名以上领军型人才（团队）和1300名硕士以上人才，新兴产业人才引进数量占比达到60%以上。加快“两化融合”步伐，推动300家企业转型升级。继续实施“千百亿工程”，力争五大主导产业销售突破4000亿元，新增1家销售超百亿元企业。

　　加快农业转型升级。大力发展优质粮油、健康肉奶、特色水产、高效园艺、生态经济林业和观光农业等六大产业。力争创成2个以上省级农业产业园，建成现代农业园区20万亩，新增高效农业22万亩、设施农业8万亩、农业龙头企业20家以上。综合农业机械化水平达80%以上。认真落实中央1号文件精神，完成水利投资30亿元，加快农村水利现代化建设，推进农村河道疏浚整治，全面完成小型水库除险加固。积极发展农业电子商务，建立农产品网上销售平台。大力发展生态农业、创意农业和品牌农业，新增无公害、绿色、有机农产品50个，抓好“菜篮子”工程，确保农产品安全有效供给。

　　实现开放型经济新发展。大力推动境外上市、增发、并购等利用外资方式，力争引进10家以上世界500强企业的分支机构，引进一批行业领先的重大外资项目、研发中心和地区销售中心。继续把招引央企作为重点，引进一批超10亿元项目，总投资超过100亿元。吸纳民资350亿元，增长35%以上。推进开发区转型升级，加快各类创新要素向开发区集聚。发挥18个省以上出口基地（企业）示范作用，促进外贸转型升级。大力发展国际服务贸易，鼓励有实力的企业 “走出去”。进一步发挥外事、侨务、对台事务在扩大开放中的积极作用。

　　（三）彰显山水花园特色，满足人民群众的生态宜居需求

　　坚持统筹城乡发展，全面开放建筑市场，强化规划、设计和施工中的人性化元素，全市完成城建投资560亿元。完善城市功能。南徐新城投入100亿元，商务A区全面交付，商务B区全面启动，职工文体中心全面竣工，主次干道全部建成，高铁站场、体育会展中心主体竣工，花园城基本成型；北部滨水区投入27亿元，基础设施全面完成，启动西津湾、文化艺术中心和金山湖旅游配套工程，滨江风貌初显；官塘新区投入50亿元，实施安置房项目及路网工程，拉开基本框架；老城棚户区改造投入10亿元，改造所有“城中村”；丹徒新城投入35亿元，加速与主城无缝对接；大港滨江产业新城投入100亿元，完善新区功能；加快各辖市城区、11个新市镇和50个新社区建设，推进“万顷良田”工程，促进城乡一体化发展。实施沪宁高速公路镇江收费站南移，编制城市轨道交通线网规划，完成泰州大桥及东接线、338省道镇江段等主体工程，开工建设镇丹高速、扬中四桥及接线等项目。

　　提升人居质量。加快更新改造，提升城市品质，争创中国人居环境奖。全面启动“绿化美化家园”工程，因地制宜种花种草、种植彩色苗木，建设花园式工厂、社区和学校。完善住宅小区综合配套设施建设。完成金山水厂改扩建和区域供水工程。大力推动“智慧镇江”建设。发放市民卡100万张，完成44万户居民电表智能化改造。认真解决群众反映强烈的停车难、行车难问题，优先发展公共交通，新辟和优化公交线路15条，更新一批公交车辆，新增各类停车泊位1万个；推进城市主干道智能交通系统建设，切实提高道路通行能力。

　　加强生态建设。继续深化国家生态市创建，丹阳、句容、扬中和丹徒创成国家生态县，全市力争通过国家级考核验收。开展国家生态园林城市创建，全面完成“青山绿水”工程，绿化造林5万亩。全力整治群众反映强烈的市区“黑臭河”问题，完成市区河道241个排污口整治，雨污分流管网全部接通。防治工业废气污染、餐饮业油烟污染、机动车尾气超标排放和工地扬尘污染，确保空气质量优良率保持在90%以上。坚持低碳发展，狠抓节能减排，严格环保执法，淘汰落后设备、技术和产能。切实保护饮用水源地，加强对农业面源污染和生活污染的监管。坚持最严格的耕地保护制度，提高资源集约利用水平。

　　优化城市管理。落实大城管机制，夯实基层基础工作，严格落实管理责任。实施市容市貌精细化整治，重点解决群众投诉较多的露天菜市场问题，新建14个、改造5个室内菜市场。加大违法设施、违章建筑拆除力度。狠抓建成区道路一体化保洁、渣土运输及建设工地管理。加快完善市政公用配套设施，推进生活垃圾分类收集与处理，新建4座压缩式垃圾转运站。

　　（四）加快发展文体事业，满足人民群众的精神文化需求

　　全力推进文明创建。以争创全国文明城市为龙头，深入开展群众性精神文明创建活动，加强未成年人思想道德建设，完善社会信用体系，不断提升市民素质，打造“大爱镇江”和志愿者服务等特色品牌。依法保护历史文化遗产。加快推进全国文明城市创建硬件达标，建成市科技馆和档案馆。

　　扎实推进文体惠民。开展“欢乐家园”和“我们的节日”系列公益文化活动，举办“2011镇江音乐季”。推进文化信息资源共享工程，建设一批特色文化广场、图书馆和文化站室。大力开展全民健身活动，城乡体育健身设施覆盖率超过90%，每万人拥有健身点5个以上，经常参加体育锻炼的人数超过30%。

　　做大做强文体产业。重点发展文化创意、影视演艺、旅游文化和出版印刷等四大文化产业。深化文化体制改革，加快提升市文广集团、报业集团实力，力争吸引1-2个全国知名的文化企业落户，完成一批文化设施建设。加快发展体育产业，构建以市体育会展中心为核心的产业链。

　　（五）全面加强社会建设，满足人民群众的公共服务需求

　　优化教育卫生服务。深入贯彻教育改革发展实施意见，扎实开展国家学前教育体制改革试点，积极推进义务教育优质均衡发展，实施东方技校等一批职校整合迁建，完成“校舍安全工程”19.4万平方米，支持在镇高校发展，办好人民满意的教育。完成市二院整体迁建、精神卫生中心异地新建和妇幼保健院整合，建成市一院内科医技楼和中心血站综合楼。全面完成国家公立医院改革试点任务。

　　提供安全保障服务。加强社会治安综合治理，依法严厉打击各类犯罪活动，巩固“平安镇江”创建成果。启动“六五”普法，建设“法治镇江”。完善公共突发事件应急机制，严格落实安全生产和食品药品安全责任，杜绝重大事故发生，确保群众生命财产安全。创新特殊人群和外来人口服务管理体系，严格规范小旅馆等“九小场所”管理。启用新人民来访接待中心，强化信访大格局建设，健全“大调解”机制，深入推进社会矛盾化解。

　　延伸社会公益服务。推进新型社区建设，完善人口公共服务体系。依法管理宗教事务，加强民族团结进步事业。加强国防教育和人民武装工作，深化军民共建，力争全国双拥模范城“六创”成功。继续开展对口支援。认真做好妇女儿童、残疾人等工作。支持工会、共青团、妇联、侨联和科协等人民团体充分发挥桥梁纽带作用。

　　（六）强化政府自身建设，满足人民群众的利益表达需求

　　新形势、新任务呼唤建设现代服务型政府，更要求全体公务人员实时维护广大群众的合法权益。

　　恪守一心为民的职责。人民政府必须对人民高度负责。全体公务人员将始终带着责任、带着使命、带着感情履职，自觉接受人大法律监督、工作监督和政协民主监督，主动接受群众监督和舆论监督；倍加珍惜来之不易的发展局面，倍加珍惜为民干事的广阔舞台，倍加珍惜自己人生的宝贵时光，时刻把群众的安危冷暖放在心上，时刻把维护群众的利益抓在手上，为人民服务、让人民放心、使人民满意。

　　锤炼锐意创新的品格。工作中多一些开放、少一些保守，多一些鼓励、少一些指责，始终保持争创一流的志气、敢为人先的锐气、求新思变的勇气。创新发展思路，提升转型升级的能力和水平；坚持走群众路线，创新工作方法，提高维护群众合法权益的实效性。

　　浓厚真抓实干的氛围。倡导“为民多吃苦、为民作奉献、为民埋头干”的风气，敢于负责、勇挑重担、真抓实干，始终把重点放到推进强市富民上，把精力集中到解决具体问题上。对群众合理的利益诉求，尤其要有决心、有恒心、有耐心加以解决，以真心换民心、以诚意得民意、以毅力聚民力，形成心心相印、同心干事的生动局面。

　　弘扬高效清廉的作风。做事雷厉风行，工作干净利落，以高效率保证高质量，以快节奏推动大发展。认真落实党风廉政建设责任制，深化工程招标、政府采购等重点领域的廉政建设，推进公共资源市场化配置。切实加强审计监察，严格控制公用经费和一般性支出，将有限的财力更多地投向民生，始终保持勤政为民、务实清廉的形象。

　　各位代表，形势催人奋进，前景令人期待！让我们在中共镇江市委的坚强领导下，紧紧依靠全市人民，为把镇江建设成为产业发达城市、创业创新城市、山水花园城市、幸福和谐城市而努力奋斗！
